# Moderate Dietary Cannabidiol Enhances Growth, Restructures Gut Microbiota, and Bolsters Environmental Stress Resilience in *Litopenaeus vannamei*

**DOI:** 10.3390/antiox15040475

**Published:** 2026-04-10

**Authors:** Jingwei Liu, Qian Lin, Jianchao Lu, Tianwei Jiang, Yukun Zhang, Weilong Wang

**Affiliations:** 1 China-ASEAN Belt and Road Joint Laboratory on Mariculture Technology (Shanghai), Shanghai Ocean University, Shanghai 201306, China; 2 Centre for Research on Environmental Ecology and Fish Nutrition of the Ministry of Agriculture, Shanghai 201306, China; 3 National Demonstration Center for Experimental Fisheries Science Education, Shanghai Ocean University, Shanghai 201306, China; 4 Hunan Provincial Key Laboratory of the Traditional Chinese Medicine Agricultural Biogenomics, Changsha Medical University, Changsha 410219, China; 5 Institute of Bast Fiber Crops, Chinese Academy of Agricultural Sciences, Changsha 410205, China; 6 Laboratory of Aquatic Animal Nutrition, Faculty of Fisheries, Kagoshima University, Kagoshima 890-0056, Japan

**Keywords:** cannabidiol, *Litopenaeus vannamei*, intestinal microbiota, stress resilience, antioxidant capacity, growth, EPA, DHA

## Abstract

Intensive aquaculture induces severe environmental stress and disease susceptibility in Pacific white shrimp (*Litopenaeus vannamei*). Cannabidiol (CBD) offers significant potential as a bioactive stress-mitigating additive. This study evaluated the effects of dietary CBD supplementation (0, 10, 20, 40, and 80 mg/kg) on the growth, intestinal microecology, and stress tolerance of juvenile *L. vannamei* over an 8-week feeding trial, followed by a combined chronic ammonia and acute hypoxia challenge. Moderate CBD supplementation (10–40 mg/kg) significantly promoted growth, minimized feed conversion ratios, and enriched muscle eicosapentaenoic (EPA) and docosahexaenoic acids (DHA). Furthermore, CBD restructured the intestinal microbiota by suppressing opportunistic pathogens and enriching beneficial taxa. Under combined stress, moderate CBD prolonged the median lethal time (LT_50_) by up-regulating hypoxia-inducible factor 1-alpha (*hif-1α*) and heat shock protein 70 (*hsp70*) transcription and boosting systemic antioxidant capacity to neutralize lipid peroxidation. Conversely, the highest dose (80 mg/kg) induced metabolic exhaustion and hepatopancreatic toxicity, evidenced by drastically elevated serum transaminases and diminished stress tolerance. Conclusively, dietary CBD exerts a classic biphasic effect in *L. vannamei*. Inclusion at 10–40 mg/kg safely promotes the best comprehensive effects on growth, immune homeostasis, and environmental resilience within the concentration range tested in this study, whereas excessive administration provokes severe metabolic burden, highlighting the critical need for strict dosage regulation.

## 1. Introduction

The rapid expansion of the aquaculture sector is a direct response to the growing global demand for high-quality animal protein [1]. Among farmed species, the Pacific white shrimp (*Litopenaeus vannamei*) has become the dominant contributor to global crustacean production, owing to its rapid developmental cycle, robust euryhaline traits, and capacity for high-density cultivation [2]. However, the shift towards hyper-intensive farming has introduced significant ecological and physiological challenges, such as water quality instability [3], ammonia nitrogen accumulation [4], and dissolved oxygen fluctuations [5]. These stressors collectively compromise the immune competence of shrimp, increasing their susceptibility to opportunistic pathogens and thereby threatening the profitability and sustainability of the industry [6,7]. Traditionally, the industry has relied upon broad-spectrum antibiotics and synthetic chemotherapeutants to manage these risks; however, the resulting emergence of antimicrobial resistance and stringent international regulations on chemical residues have necessitated the search for sustainable, bioactive alternatives [8,9,10].

Phytogenic compounds and nutraceuticals have emerged as promising candidates for improving the health and welfare of aquatic organisms [11,12]. Cannabidiol (CBD), a predominant non-psychoactive phytocannabinoid derived from the industrial hemp plant (*Cannabis sativa* L.), has recently gained significant scientific interest for its extensive range of therapeutic properties [13,14]. Unlike Δ^9^-tetrahydrocannabinol (THC), CBD is characterized by a high safety margin and does not exhibit addictive potential [15]. Extensive biomedical literature has firmly established its roles in modulating inflammation [16,17], neutralizing oxidative stress [18,19], and regulating immune responses in various mammalian models [20,21].

In recent years, the application of CBD in aquatic sciences has advanced from preliminary model organisms to commercially significant species [22]. Recent evidence demonstrates that waterborne administration of CBD can effectively mitigate transport-induced acute stress and behavioral anomalies in ornamental fishes [23]. Furthermore, dietary supplementation with CBD or cannabis derivatives has been documented to significantly enhance the welfare of Nile tilapia (*Oreochromis niloticus*) by ameliorating aggressive behavior, positively regulating hematological profiles, and boosting overall metabolic responses [22,24,25,26]. Similar physiological improvements have also been reported in marine teleosts, such as the large yellow croaker (*Larimichthys crocea*) [27]. Mechanistically, these benefits in finfish are frequently linked to the regulatory interaction between CBD and the endocannabinoid system or homologous neurological signaling networks [28].

For invertebrates such as *L. vannamei*, the polyphenol-like structure of CBD—particularly its rich hydroxyl groups—confers exceptional radical-scavenging capabilities [29,30]. This structural advantage highlights its unexplored potential to preserve physiological homeostasis and alleviate oxidative damage in shrimp subjected to environmental stressors. Beyond these direct biochemical benefits, CBD may also exert systemic protective effects via the host’s gut-microbiome axis. The gastrointestinal tract serves as the primary organ for nutrient assimilation and the first line of defense against aquatic pathogens [31,32]. While mammalian models have indicated that CBD can interact with the gut microbiome to alleviate intestinal inflammation [33,34], its capacity to modulate the intestinal microecosystem in aquatic invertebrates remains entirely unknown.

The current study aimed to systematically evaluate the impact of dietary CBD supplementation on the growth trajectory, feed utilization, muscle nutritional profile, and intestinal microbiota architecture of *L. vannamei*. Furthermore, we subjected the shrimp to a combined chronic ammonia and acute hypoxia challenge to clarify the role of CBD in bolstering environmental stress tolerance. Crucially, by setting a wide range of inclusion gradients (0, 10, 20, 40 and 80 mg/kg diet, based on validated effective doses in aquaculture species [25,26,27]), this study also sought to elucidate the dose-dependent biphasic effects of CBD, aiming to identify the most effective dietary dosage, assess the physiological risks associated with excessive administration, and provide empirical support for the application of CBD in sustainable intensive shrimp aquaculture.

## 2. Materials and Methods

### 2.1. Ethics Statement

The handling and culture of the animals used in this research study were carried out in compliance with the guidelines established by the Animal Ethics Committee of Shanghai Ocean University (Shanghai, China), following the approved protocol numbers SHOU-DW-2020-017 (Approved on 15 March 2020).

### 2.2. Feed Ingredients and Diets Formulation

Five isonitrogenous (42% crude protein) and isolipidic (6% crude lipid) experimental diets were formulated to meet the nutritional requirements of juvenile *L. vannamei*. Using fish meal, meat meal, soybean meal, peanut meal, and corn gluten meal as protein sources, and fish oil, soybean oil, and soybean lecithin as lipid sources, a basal diet was formulated. The functional additive used in this study was dried hemp (*C. sativa* L.) leaf powder, which contained approximately 5% active CBD and was kindly provided by the Institute of Bast Fiber Crops, Chinese Academy of Agricultural Sciences (Changsha, China).

To achieve the target active CBD levels of 0, 10, 20, 40, and 80 mg/kg in the final diets (designated as Group A, B, C, D, and E, respectively), the hemp leaf powder was supplemented at exactly 0%, 0.02%, 0.04%, 0.08%, and 0.16% of the total diet, respectively. Corresponding reductions in α-cellulose content were made across all diets to maintain equal protein and lipid levels, ensuring the only variable among the five experimental diets was the inclusion level of CBD.

The CBD inclusion levels were set based on published effective doses in farmed fish [25,26,27], our core objective to clarify dose-dependent effects of CBD in shrimp, and species differences between crustaceans and teleosts, to ensure both the capture of significant biological effects and experimental safety. Due to the minimal inclusion levels of CBD (≤0.16% of diet), its contribution to the overall protein and lipid content was negligible, and the adjustments in cellulose effectively maintained isonitrogenous and isolipidic conditions across all diets.

All dry ingredients were ground through an 80-mesh sieve, weighed accurately according to the formulation, and thoroughly mixed in a stepwise manner. After adding fish oil, soybean lecithin, and an appropriate amount of water, the mixture was pelleted into 1.5 mm diameter feed pellets using a pelletizer. The pellets were immediately dried to a moisture content of 10% at low temperature, sealed in vacuum bags, and stored at −20 °C until use. The composition and nutritional levels of the basal diet are shown in Table 1.

### 2.3. Animals, Experimental Procedure, and Conditions

The feeding trial was conducted at the Binhai Aquaculture Base of Shanghai Ocean University. A total of 675 juvenile *L. vannamei* (purchased from Longyuan Seedling Company, Nantong, China) with uniform size, healthy constitution, and an average body weight of 0.52 ± 0.04 g were selected and randomly distributed into 15 indoor recirculating tanks (height: 117 cm, radius: 50 cm, spatially randomized), with 45 shrimp per tank. Each tank was covered with a net to prevent shrimp from jumping out. Shrimp were fed to satiation four times daily (at 07:35, 12:35, 17:35, and 22:35). Feed residues were collected 2 h after each feeding (this time point was set to ensure complete recovery of settled uneaten feed with minimal nutrient leaching into water) to monitor feeding activity, and daily feed rations were adjusted accordingly.

The 8-week feeding trial was conducted under controlled conditions: water temperature 27–29 °C, salinity 10‰, pH 7.8–8.5, with continuous aeration maintaining dissolved oxygen above 7 mg/L. The system operated under natural photoperiod with 20% water exchange every 3 days, while ammonia nitrogen was kept below 0.2 mg/L and water transparency was maintained at 20–40 cm throughout the experimental period.

At the end of the 8-week feeding trial, all samples for basal growth performance, physiological and biochemical analysis, and intestinal microbiota detection were collected first. The remaining healthy shrimp with uniform size in each replicate tank were fasted for 24 h and then used for the subsequent environmental stress challenge experiment.

### 2.4. Chronic Ammonia-Nitrite Stress

After the completion of basal sample collection, a 4-day combined ammonia and nitrite stress test was conducted. Healthy shrimp from each treatment were exposed to water containing 2 mg/L of nitrite-nitrogen (NO_2_^−^-N) and 2 mg/L ammonia-nitrogen, maintained by adding reagent-grade NaNO_2_ and NH_4_Cl. Water quality was monitored twice daily to ensure stable stress concentrations.

### 2.5. Acute Hypoxia Stress and LDO50/LT50 Determination

Immediately following the 4-day chemical stress, an acute hypoxia challenge was performed to evaluate the resilience of the shrimp. Healthy shrimp with uniform size were randomly selected and fasted for 12 h prior to the hypoxia tolerance test. 10 shrimp from each replicate tank were transferred into a separate 20 L sealed bucket. Each replicate tank corresponded to 1 hypoxia challenge bucket, with 3 biological replicate buckets per dietary CBD treatment group, resulting in a total of 30 shrimp per treatment group for the hypoxia test.

Dissolved oxygen (DO) in the sealed buckets was allowed to decline naturally through shrimp respiration, and the DO concentration was continuously monitored using a portable dissolved oxygen meter. The time of death and the corresponding DO level for each individual shrimp were recorded precisely. Death was defined as the total cessation of pleopod movement and lack of response to mechanical stimulation. The median lethal dissolved oxygen (LDO_50_) and the median lethal time (LT_50_) for each treatment group were calculated using Probit regression analysis, which modeled the relationship between cumulative mortality and the DO concentration or logarithm of exposure time (log_10_T) for each of the 3 biological replicates per group, respectively, with the final group value presented as the mean of the 3 replicates.

The Kaplan–Meier cumulative survival curve for each dietary treatment group was plotted based on the exact death time of all 30 individual shrimp (merged from 3 biological replicate buckets) in the group.

### 2.6. Sample Collection and Biochemical Analyses

At the end of the 8-week feeding trial and prior to the initiation of the combined ammonia-nitrite and hypoxia stress challenge, fecal samples were collected 1.5 h post-feeding using siphoning during the final two weeks of the culture period [35]. This time point was selected as the excretion window for *L. vannamei*, ensuring feces with intact nutritional components for accurate apparent digestibility analysis. All collection operations adopted low-disturbance protocols: fecal collection was performed by gentle siphoning along the tank wall without touching shrimp, and all sampling was completed within 5 min per tank at a fixed time, which avoided acute stress responses of shrimp caused by human interference. Collected feces were transferred to 10 mL centrifuge tubes and stored at −20 °C for apparent digestibility analysis. Upon trial completion, shrimp were fasted for 24 h before harvest. Final body weight and survivability (SV) were recorded. In each tank, 12 intermolt shrimp were randomly selected and anesthetized (100 mgL^−1^ tricaine methane sulfonate; MS222; Sigma-Aldrich, St. Louis, MO, USA): three were used for whole-body composition analysis; the rest were used for hemolymph collection (withdrawn from the pericardial cavity using sterile 1 mL syringes). The hemolymph was then stored in 2 mL tubes at −80 °C for biochemical/enzymatic assays. Muscle tissues and intact hepatopancreas were dissected into 5 mL centrifuge tubes and preserved at −80 °C for digestive/antioxidant enzyme analysis. For gut microbiome analysis, three shrimp per recirculating tank were surface-sterilized with 75% alcohol, aseptically dissected, and the intestines were collected. After rinsing with sterile saline, the intestines were transferred to cryovials and stored at −80 °C.

Proximate composition (crude protein, crude lipid, moisture, and ash contents) of experimental diets, shrimp body samples, and feces was determined according to the standard methods of the Association of Official Analytical Chemists [36]. Briefly, moisture content was measured by oven-drying at 105 °C to a constant weight. Crude protein was analyzed by the Kjeldahl method using an automatic Kjeldahl analyzer (2300 Auto-analyzer, Foss Tecator, Höganäs, Sweden). Total lipids were extracted with chloroform-methanol (2:1, *v*/*v*) containing 0.01% BHT following the methods of Cejas [37], then methylated with 14% BF_3_ in methanol [38]. Ash content was determined by incinerating in a muffle furnace at 550 °C for six hours. Fecal Y_2_O_3_ content was quantified using ICP-MS (ICAPQc, SN03019R, ThermoFisher (Waltham, MA, USA)).

Fatty acids were extracted, and fatty acid methyl esters (FAMEs) were prepared according to the ISO 5509 method [39]. Briefly, total lipids were first extracted from samples via Soxhlet extraction with petroleum ether at 60 °C for 6 h. The extracted lipids were then saponified with 0.5 mol/L potassium hydroxide-methanol solution at 60 °C for 20 min, followed by esterification with 14% boron trifluoride-methanol solution at 70 °C for 15 min to obtain FAMEs, which were finally extracted with n-hexane and dehydrated with anhydrous sodium sulfate.

FAMEs were subsequently analyzed using an Agilent 7890 capillary gas chromatograph (GLC) equipped with a flame ionization detector (FID; Agilent Technologies, Santa Clara, CA, USA). Separation was performed on a Supelco SP-2560 capillary column (30 m × 0.25 mm I.D., 0.5 μm film thickness; Supelco, Bellefonte, PA, USA). The chromatographic conditions were set as follows: injector temperature 210 °C, FID temperature 250 °C; carrier gas high-purity N_2_ at a constant flow rate of 1.0 mL/min; split ratio 10:1; oven temperature programmed from 180 °C (held for 2 min) to 250 °C at a rate of 4 °C/min, and held at 250 °C for 10 min.

Qualitative and quantitative analyses of fatty acids were performed using a FAME Mix standard (Cat. No. 47885-U, Supelco, Bellefonte, PA, USA). Target fatty acids were identified by matching the retention time of sample peaks with those of the standard, and the relative content of each fatty acid was calculated by the peak area normalization method, expressed as a percentage of the total fatty acids.

Digestive enzymatic activities and biochemical assays were analyzed using the hepatopancreas from four randomly selected shrimp in total from the three replicate tanks of each dietary treatment. The hepatopancreas was homogenized with sterilized shrimp saline (0.85%, 1:9 *w*/*v*) and centrifuged (8000 rpm, 10 min, 4 °C). The supernatant was used for following analyses: protease activity was determined by Folin method (GB/T 23527.1-2023) [40]; lipase activity, amylase activity, Na^+^, K^+^-ATPase activity, total antioxidant capacity (T-AOC), malondialdehyde (MDA) content, and catalase (CAT) activity, glutathione peroxidase (GSH-Px) activity, and peroxidase (POD) activity were assayed using commercial kits (Nanjing Jiancheng Bioengineering Institute (Nanjing, China)); serum parameters including glucose (GLU), triglycerides (TG), alkaline phosphatase (ALP), acid phosphatase (ACP), lysozyme (LZM), alanine aminotransferase (ALT), and aspartate aminotransferase (AST), as well as serum T-AOC, CAT activity, and MDA content, were measured with corresponding kits following manufacturer’s protocols (Nanjing Jiancheng Bioengineering Institute). To minimize subjective bias, the personnel involved in the outcome assessment and data analysis were blinded to the dietary group allocations.

### 2.7. RNA Isolation and qPCR Analysis

For gene expression analysis, 1 shrimp at the terminal moribund state was collected from each hypoxia challenge replicate bucket when the cumulative mortality of the bucket reached 100% (3 shrimp per dietary treatment group, corresponding to 3 independent biological replicates). The sampled shrimp was the last surviving individual in the bucket, with weak pleopod movement and minimal response to standardized mechanical stimulation, immediately before meeting the irreversible death criteria defined in Section 2.5. Hepatopancreas tissues were dissected immediately after sampling, snap-frozen in liquid nitrogen, and stored at −80 °C for subsequent RNA extraction.

Total RNA was extracted from the hepatopancreas of size-matched shrimp using Total RNA Extraction Kits (Tiangen, Beijing, China). RNA integrity was verified by 1% agarose gel electrophoresis, and concentration/purity was determined via NanoDrop 2000 spectrophotometer (Thermo Scientific, Waltham, MA, USA). Subsequent cDNA synthesis employed PrimeScript™ RT reagent Kits (Takara Bio Inc., Kusatsu, Shiga, Japan). Gene-specific primer sequences for heat shock protein 70 (*hsp70*), cysteine-aspartic acid protease 3 (*caspase 3*), hexokinase (*hk*), hypoxia-inducible factor 1-alpha (*hif-1⍺*), and lactate dehydrogenase (*ldh*) are shown in Table 2. Quantitative PCR amplification followed Wang et al. [41], with relative gene expression calculated using the 2^−ΔΔCt^ method [42], with β-actin as the reference gene.

### 2.8. DNA Extraction, Library Preparation, and High-Throughput Sequencing Data Analysis

Genomic DNA was extracted from intestinal samples using the EZNA^®^ Soil DNA Kit (Omega Bio-tek, Norcross, GA, USA) following the manufacturer’s protocol. The hypervariable V3–V4 region of the bacterial 16S rRNA gene was amplified via PCR with extracted DNA as template. Amplification products were purified by electrophoresis on agarose gels and quantified using a Qubit^®^ 4.0 Fluorometer (Thermo Fisher Scientific). Sequencing libraries were constructed with the NEXTFLEX^®^ Rapid DNA-Seq Kit (Bioo Scientific (Austin, TX, USA)), involving adapter ligation, size selection with magnetic beads, and PCR enrichment. Final libraries were sequenced on the Illumina (San Diego, CA, USA) (NovaSeq 6000) platform. Raw sequence data have been uploaded to NCBI (PRJNA1434381).

Raw paired-end reads were quality-filtered and adapter-trimmed using fastp (v0.23.2). Overlapping reads were merged with FLASH (v1.2.11). Operational Taxonomic Units (OTUs) were clustered at 97% sequence similarity using USEARCH (v11.0). Sequences annotated as chloroplasts or mitochondria were removed. Taxonomic classification was performed against the SILVA (v138) 16S rRNA reference database using the RDP classifier (v2.13) with a confidence threshold of 0.8. Microbial community composition, including α-diversity indices and β-diversity metrics, was analyzed. Linear Discriminant Analysis Effect Size (LEfSe) was performed to identify the microbial biomarkers exhibiting significant differential abundance across groups (LDA score > 3.0). The differential abundance of specific bacterial taxa at the genus level was further validated using the Kruskal–Wallis H test. Functional profiles of bacterial communities were predicted using PICRUSt2 based on the Clusters of Orthologous Genes (COG) database.

### 2.9. Calculations

The performance indices were calculated as follows:

Weight Gain Rate (WGR, %) = [(Wₜ − W_0_)/W_0_] × 100

Survivability (SV, %) = (Nₜ/N_0_) × 100

Feed intake (FI, %day) = [W_f_/((Wₜ × N_0_/N_t_ + W_0_)/2)]/t × 100

Feed Conversion Ratio (FCR) = W_f_/(Wₜ − W_0_)

Specific Growth Rate (SGR, % d^−1^) = [ln(Wₜ) − ln(W_0_)]/t × 100

Apparent Dry Matter Digestibility (ADDM, %) = [1 − (B_1_/B_2_)] × 100

Apparent Nutrient Digestibility (AND, %) = [1 − (A_1_/A_2_) × (B_1_/B_2_)] × 100

where Wₜ: Final mean body weight (g), W_0_: Initial mean body weight (g), W_f_: Total dry matter intake (g), t: Experimental duration (days), Nₜ: Final number of shrimp per tank, N_0_: Initial number of shrimp per tank, A_1_: Nutrient concentration in feces (%), A_2_: Nutrient concentration in diet (%), B_1_: Y_2_O_3_ concentration in diet (%), B_2_: Y_2_O_3_ concentration in feces (%)

### 2.10. Statistical Analysis

All experimental results are expressed as means ± standard deviation (mean ± SD). For growth performance and survival rate, the experimental unit was the tank (*n* = 3 per treatment). For biochemical parameters, fatty acid composition, and enzyme activities, the experimental unit was the individual shrimp, with four shrimp randomly selected per treatment (*n* = 4 per treatment). Data normality and homogeneity of variances were verified prior to analysis. Statistical analyses were performed using SPSS 25.0 (IBM Corp., Armonk, NY, USA). Significant differences among treatment groups were determined by one-way analysis of variance (ANOVA), followed by Duncan’s multiple range test for post hoc comparisons when significant effects (*p* < 0.05) were detected. For microbiota data, non-parametric tests (e.g., Kruskal–Wallis) were applied where data did not meet parametric assumptions. Cumulative survival curves were generated using the Kaplan–Meier method in GraphPad Prism 9.0.

To systematically evaluate the potential internal relationships between macro-phenotypes and micro-physiological changes, Pearson correlation analysis was conducted among the core parameters of growth performance, digestive enzymes, antioxidant status, stress-related genes, and environmental stress resistance. The correlation coefficient (*r*) matrix and corresponding *p*-values were calculated and visualized using GraphPad Prism 9.0. Statistical significance for the correlation was defined as *p* < 0.05.

## 3. Results

### 3.1. Growth Performance, Feed Utilization, and Whole-Body Proximate Composition

The effects of dietary CBD on the growth performance, feed utilization, and survival of *L. vannamei* are presented in Table 3. The IBW showed no significant differences among the treatments (*p* > 0.05). At the end of the feeding trial, shrimp fed diets supplemented with varying levels of CBD exhibited significantly higher FBW, WGR, and SGR compared to the control group (*p* < 0.05). However, no significant differences in these growth parameters were observed among the different CBD-supplemented groups (*p* > 0.05).

Regarding feed utilization, the FCR exhibited a distinct U-shaped dose–response pattern. The FCR was significantly reduced in groups fed 10 to 40 mg/kg CBD compared to the control, reaching its lowest value of 1.68 in Group D (*p* < 0.05). However, when the CBD dosage increased to 80 mg/kg (Group E), the FCR deteriorated to 1.86, showing no significant difference from the control group (*p* > 0.05). Conversely, FI progressively increased, peaking significantly in Group E. Furthermore, the SV was significantly enhanced by moderate CBD supplementation, maximizing at 80.74% in Group D (*p* < 0.05). Notably, in the highest dosage group, the SV declined to 74.81%, which was not significantly different from the control group (*p* > 0.05).

The whole-body proximate composition of the shrimp fed the experimental diets is summarized in Table 4. Dietary CBD supplementation did not significantly affect the moisture, crude protein, and ash contents of the whole shrimp across all treatments (*p* > 0.05). Nevertheless, the total lipid content was significantly influenced by the dietary treatments. Compared to the control group (1.52%), the total lipid content remained relatively stable in the low-dose groups but significantly increased to its highest level (1.61%) in Group D (40 mg/kg) (*p* < 0.05). Interestingly, in the high-dose group (Group E, 80 mg/kg), the total lipid content significantly decreased to 1.48%, representing the lowest value among all experimental groups (*p* < 0.05).

### 3.2. Muscle Fatty Acid Profile

We further analyzed the muscle fatty acid profile of *L. vannamei* fed with varying levels of CBD (Table 5). Overall, the total saturated fatty acid (ΣSFA) content remained highly stable across all dietary treatments, showing no significant differences among the groups (*p* > 0.05).

Conversely, dietary CBD supplementation significantly altered the monounsaturated (ΣMUFA) and polyunsaturated fatty acid (ΣPUFA) profiles. The total ΣMUFA content was significantly reduced in all CBD-treated groups compared to the control group (*p* < 0.05). This reduction was primarily driven by a significant decrease in oleic acid (C18:1), which dropped from 20.13% in the control group to its lowest level of 18.69% in Group B (10 mg/kg) (*p* < 0.05).

Most notably, CBD supplementation significantly promoted the accumulation of ΣPUFAs in the shrimp muscle, with the most pronounced effect observed at the lowest inclusion level. The total ΣPUFA content reached a maximum of 50.70% in Group B (*p* < 0.05). This overall increase was heavily attributed to the significant enrichment of n-3 PUFAs. Specifically, the levels of eicosapentaenoic acid (EPA, C20:5n-3) and docosahexaenoic acid (DHA, C22:6n-3) peaked significantly in Group B at 15.22% and 12.62%, respectively, both of which were significantly higher than those in the control group (13.47% and 10.74%) (*p* < 0.05). Although the contents of n-3 PUFA, EPA, and DHA slightly declined as the CBD dosage further increased from 20 to 80 mg/kg (Groups C, D, and E), they generally remained elevated compared to the control. Meanwhile, the total n-6 PUFA content showed slight fluctuations among the treatments, largely reflecting the variations in linoleic acid (LA, C18:2n-6), which was significantly minimized in Group B.

### 3.3. Hepatopancreatic Digestive Enzyme Activities and Apparent Digestibility

The effects of dietary CBD supplementation on hepatopancreatic digestive enzyme activities and the apparent digestibility of nutrients in *L. vannamei* are presented in Table 6. Regarding the digestive enzymes, dietary CBD significantly stimulated the secretion and activity of key metabolic enzymes compared to the control (*p* < 0.05). Specifically, the hepatopancreatic protease activity was significantly elevated in all CBD-supplemented groups compared to Group A, with no significant differences observed among the varying CBD dosages (*p* > 0.05). Amylase activity exhibited a dose-dependent increase, peaking significantly in Group D (40 mg/kg) (*p* < 0.05). Interestingly, the activities of lipase and Na^+^, K^+^-ATPase followed an inverted U-shaped trend. Both enzymes reached their maximum activities in Group C (20 mg/kg), which were significantly higher than those of the control group (*p* < 0.05). However, as the CBD dosage further increased to 80 mg/kg, the activities of lipase and Na^+^, K^+^-ATPase declined to levels that showed no significant difference from the control group (*p* > 0.05).

Consistent with the trends observed in hepatopancreatic enzymatic activities, the apparent digestibility of nutrients was notably enhanced by moderate CBD inclusion. The apparent digestibility of dry matter and crude protein reached their peak values in Group C (20 mg/kg) at 84.83% and 89.31%, respectively, both of which were significantly higher than those in the control group (*p* < 0.05). Similarly, the apparent digestibility of crude lipid was significantly improved in both Group B (10 mg/kg) and Group C (20 mg/kg) compared to Group A (*p* < 0.05). Consistent with the enzymatic decline at high dosages, when the CBD supplementation reached 80 mg/kg (Group E), the apparent digestibility parameters for dry matter, crude protein, and crude lipid all decreased, showing no significant differences from the control group (*p* > 0.05).

### 3.4. Serum Biochemical Parameters

The activities of serum transaminases, which serve as indicators of hepatopancreatic health, were significantly affected by dietary CBD (Table 7). The ALT activity remained stable across the control and low-to-moderate dosage groups (*p* > 0.05). However, ALT activity exhibited a sharp and significant increase in the high-dose group (Group E, 80 mg/kg), reaching more than double the activity level of the control group (*p* < 0.05). Conversely, AST activity was significantly reduced only in Group B (10 mg/kg) (*p* < 0.05), while no significant differences were observed among the other treatments (*p* > 0.05).

Dietary CBD supplementation generally enhanced serum non-specific immune responses at moderate dosages. The LZM activity showed an increasing trend with CBD supplementation, reaching its highest level in Group D (40 mg/kg), which was significantly higher than that of the control group (*p* < 0.05). Similarly, the activities of ALP and ACP were significantly elevated in groups fed 20 and 40 mg/kg CBD compared to the control group (*p* < 0.05). When the CBD dosage increased to 80 mg/kg, the activities of LZM, ALP, and ACP declined, showing no significant differences from the control group (*p* > 0.05).

The antioxidant capacity and lipid peroxidation levels exhibited a clear dose-dependent response to CBD. The T-AOC was significantly enhanced in Groups B and C, peaking at 0.49 mM in Group C (20 mg/kg) (*p* < 0.05). However, T-AOC significantly dropped below the control level in Group E (*p* < 0.05). CAT activity peaked significantly in Group D (40 mg/kg) (*p* < 0.05) and returned to baseline levels in Group E. Consequently, the concentration of MDA, a primary marker of oxidative stress and lipid peroxidation, was significantly reduced in Groups B, C, and D compared to the control (*p* < 0.05). Notably, in the 80 mg/kg group, the MDA content rebounded to 4.85 mmol/L, which was the highest among all groups and not significantly different from the control (*p* > 0.05).

### 3.5. Hepatopancreatic Antioxidant Parameters

The effects of dietary CBD levels on the hepatopancreatic antioxidant capacity of *L. vannamei* are detailed in Table 8. Overall, dietary CBD supplementation significantly enhanced the antioxidant defense system in the hepatopancreas compared to the control (*p* < 0.05).

The T-AOC and the activity of GSH-Px exhibited similar dose–response patterns, both reaching their maximum values in Group B (10 mg/kg), which were significantly higher than those in all other treatments (*p* < 0.05). Although their activities decreased as the CBD inclusion level further increased (Groups C, D, and E), T-AOC remained significantly elevated compared to the control group (*p* < 0.05). However, the GSH-Px activity in the highest dosage group (Group E, 80 mg/kg) declined to a level that showed no significant difference from the control group (*p* > 0.05).

Similarly, the activities of CAT and POD were significantly up-regulated in the low- to moderate-dose groups (Groups B, C, and D) compared to Group A (*p* < 0.05), with both enzymes recording their peak activities in Group D (40 mg/kg). Consistent with the other antioxidant markers, when the CBD dosage increased to 80 mg/kg (Group E), the activities of CAT and POD diminished, exhibiting no significant differences from the control group (*p* > 0.05).

Consequent to the up-regulation of antioxidant enzymes, the concentration of MDA, a primary biomarker of lipid peroxidation and oxidative damage, was significantly reduced by CBD supplementation. The hepatopancreatic MDA content in all CBD-treated groups (Groups B to E) was significantly lower than that in the control group (*p* < 0.05).

### 3.6. Modulation of Intestinal Microbiota Composition by Dietary CBD

#### 3.6.1. Sequencing Quality and Alpha Diversity

**Figure 1:**
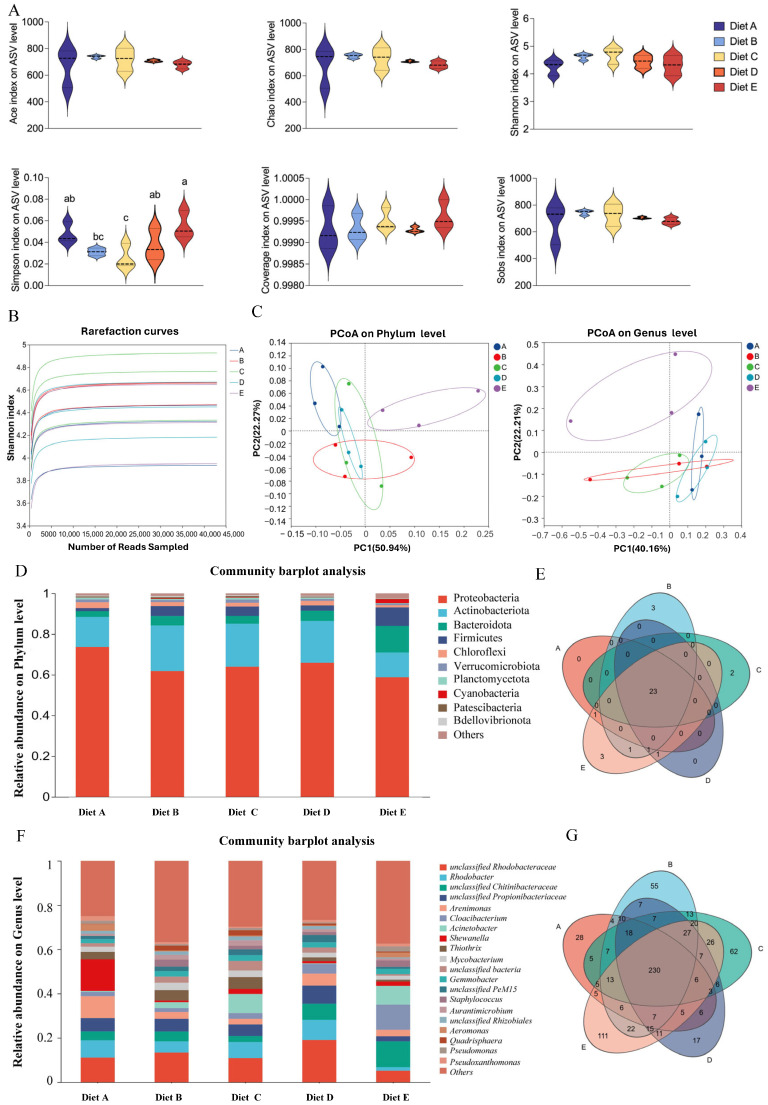
Effects of dietary cannabidiol (CBD) supplementation on the diversity and taxonomic composition of the intestinal microbiota in *L. vannamei*. (**A**) Alpha diversity indices of the intestinal microbiota, including Ace, Chao, Shannon, and Simpson indices. Different lowercase letters above the violin plots indicate statistically significant differences among dietary treatments (*p* < 0.05). (**B**) Rarefaction curves validating the sequencing depth and species richness across all samples. (**C**) Principal Coordinates Analysis (PCoA) plots illustrating beta diversity and structural variations of the microbial communities at the phylum (**left**) and genus (**right**) levels. (**D**) Community barplot analysis showing the relative abundance of dominant bacterial taxa at the phylum level. (**E**) Venn diagram displaying the shared and unique bacterial phyla among the five experimental groups. (**F**) Community barplot analysis detailing the relative abundance of dominant bacterial taxa at the genus level. (**G**) Venn diagram illustrating the shared and unique operational taxonomic units (OTUs) at the genus level. Groups A, B, C, D, and E correspond to dietary CBD inclusion levels of 0, 10, 20, 40, and 80 mg/kg, respectively.

To evaluate the impact of dietary CBD on the intestinal microecosystem of *L. vannamei*, high-throughput 16S rRNA gene sequencing was performed. The sequencing depth and species richness were first validated. As illustrated by the rarefaction curves (Figure 1B), all samples tended to reach a plateau, and the coverage index for all groups was greater than 99.9%, indicating that the sequencing depth was sufficient to capture most of the microbial diversity within the shrimp intestine. Community richness was evaluated using the Ace and Chao indices, while diversity was assessed by the Shannon and Simpson indices (Figure 1A). Regarding alpha diversity, the Simpson index showed a trend of decreasing first and then increasing with the elevation of dietary CBD supplementation. The lowest Simpson index was observed in Group C, which was significantly different from Groups A, D, and E (*p* < 0.05), but showed no significant difference compared to Group B (*p* > 0.05). Conversely, Group E recorded the highest value, which was significantly higher than those of Groups B and C (*p* < 0.05). No significant differences were observed in the other indices (*p* > 0.05).

#### 3.6.2. Beta Diversity and Community Structure

To evaluate the beta diversity and visualize the structural variations across different treatments, Principal Coordinates Analysis (PCoA) was performed at both the phylum and genus levels (Figure 1C). At the phylum level, the first two principal coordinates (PC1 and PC2) accounted for 50.94% and 22.27% of the total structural variation, respectively. The scatter plot revealed that Group E (80 mg/kg CBD) was distinctly separated from all other groups, indicating a unique and profound shift in the microbial community architecture at this dosage. The control group (Group A) showed no spatial intersection with Groups B and E but exhibited a partial overlap with Groups C and D. Data points for Groups B, C, and D overlapped with each other, suggesting a relatively high degree of structural similarity among these moderate-dose treatments. At the genus level (PC1: 40.16%, PC2: 22.21%), Group E remained clearly isolated. Group A displayed no overlap with Groups C and E. Notably, while Groups C and D did not overlap directly, they were tightly clustered in close spatial proximity. Group B appeared as a transitional cluster, overlapping simultaneously with Groups A, C, and D. Together, these spatial distributions indicate that the highest CBD dosage (Group E, 80 mg/kg) establishes a microbial community profile structurally distinct from both the control and the lower-dosage groups.

#### 3.6.3. Taxonomic Composition Profiles

At the phylum level (Figure 1D), the dominant bacterial phyla across all groups were *Proteobacteria*, *Actinobacteriota*, *Bacteroidota*, and *Firmicutes*. The Venn diagram (Figure 1E) showed a highly conserved core microbiome, sharing numerous phyla across all groups, with only a few unique phyla appearing in specific treatments. At the genus level (Figure 1F), the community was predominantly composed of *unclassified_Rhodobacteraceae*, *Rhodobacter*, *unclassified_Chitinibacteraceae*, and *unclassified_Propionibacteriaceae*. The genus-level Venn diagram (Figure 1G) revealed a shared core of 230 operational taxonomic units (OTUs) among all five groups, demonstrating that CBD treatments recruited specific, unique bacterial members to the intestinal environment while maintaining the core community.

### 3.7. Differential Taxa and Functional Profiling of the Microbiome

#### 3.7.1. Hierarchical Clustering and Abundance Patterns

**Figure 2:**
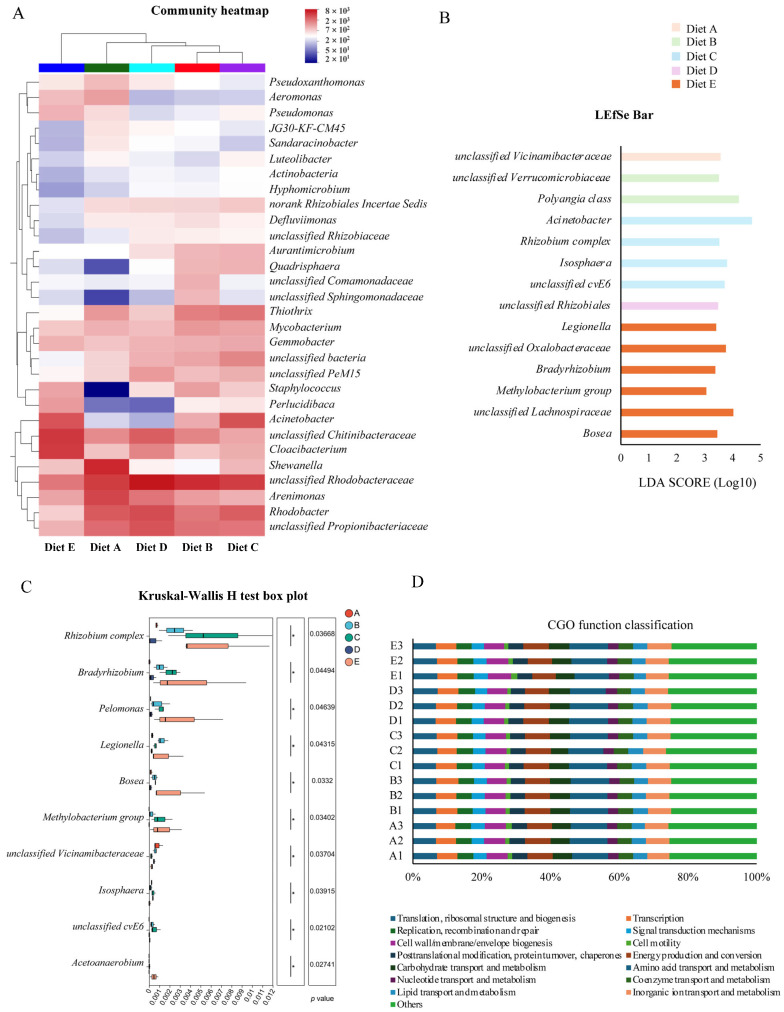
Differential abundance and functional prediction of the intestinal microbiota in *L. vannamei* across different dietary CBD treatments. (**A**) Community heatmap and hierarchical clustering of the top abundant bacterial genera. Color gradients indicate the relative abundance of specific microbial taxa. (**B**) Linear discriminant analysis Effect Size (LEfSe) identifying significantly enriched microbial biomarkers across the experimental groups (logarithmic LDA score threshold > 3.0, *p* < 0.05). (**C**) Differential abundance analysis of specific key bacterial genera evaluated using the Kruskal–Wallis H test (*, *p* < 0.05). (**D**) Predicted functional profiles of the intestinal microbiome based on the Clusters of Orthologous Genes (COG) database. Groups A, B, C, D, and E correspond to dietary CBD inclusion levels of 0, 10, 20, 40, and 80 mg/kg, respectively.

To further identify specific microbial shifts, a community heatmap at the genus level (Figure 2A) was generated to provide hierarchical clustering of the top abundant genera. The color gradients visually confirmed that CBD treatments induced distinct abundance patterns compared to the control group. Specifically, Group A exhibited a relative enrichment of taxa such as *Shewanella*, *Arenimonas*, and the opportunistic pathogen *Aeromonas*. In contrast, the low- to moderate-dose CBD treatments (Groups B, C, and D) effectively suppressed these taxa. Concurrently, these moderate dosage groups displayed a distinct enrichment of core microflora, e.g., *unclassified_Rhodobacteraceae* and *Staphylococcus*. Notably, the high-dose group (Group E, 80 mg/kg) displayed a completely divergent clustering profile. Within this group, the previously enriched *Rhodobacter* and *Rhodobacteraceae* were relatively depleted, replaced by a profound surge in *norank_f__Chitinibacteraceae* and *Cloacibacterium*. Furthermore, potentially pathogenic genera like *Pseudomonas* and *Aeromonas* showed a rebounding trend compared to the moderate-dose groups. These visual trends corroborate that while moderate CBD supplementation positively modulates the intestinal microbiota, the highest dosage group (80 mg/kg) triggers a drastic structural reconstruction in the current study.

#### 3.7.2. Identification of Specific Microbial Biomarkers

To identify the specific bacterial taxa driving the structural differences, Linear Discriminant Analysis Effect Size (LEfSe) was performed. A total of 14 discriminative features were identified as significantly enriched biomarkers based on a logarithmic LDA score threshold of >3.0 and a significance level of *p* < 0.05 (Figure 2B). Overall, dietary CBD significantly altered the microbial biomarkers in a dose-associated manner. In the low- to moderate-dose groups (Groups B, C, and D), specific taxa such as *Polyangia* class, *Acinetobacter* (the highest overall LDA score of 4.69 in Group C), and *unclassified_Rhizobiales* were significantly enriched. Notably, Group E (80 mg/kg CBD) recruited the highest number of specific biomarkers (six taxa). Within this high-dose group, *unclassified_Lachnospiraceae* (LDA = 4.02) and *unclassified_Oxalobacteraceae* (LDA = 3.76) emerged as prominently enriched clades, alongside *Bosea* and *Bradyrhizobium*. These results demonstrate that the high dosage of CBD exerts a strong selective pressure on the microecosystem, leading to an over-representation of specific microbial lineages.

#### 3.7.3. Differential Abundance of Key Genera

To further validate the taxonomic shifts, a differential abundance analysis of key genera was conducted using the Kruskal–Wallis H test (Figure 2C). The results demonstrated that dietary CBD significantly altered the relative abundances of ten specific bacterial genera (*p* < 0.05). Specifically, the control group exhibited the highest abundance of *unclassified_Vicinamibacteraceae*. In the moderate-dose CBD groups, specific taxa such as *unclassified_cvE6* and *Isosphaera* were significantly enriched. Notably, the high-dose treatment (Group E, 80 mg/kg) exerted the most profound effect, triggering the maximum accumulation of multiple distinct genera, predominantly including *Bradyrhizobium*, *Pelomonas*, and *Bosea*. These findings objectively confirm that the 80 mg/kg CBD dosage imposes a strong environmental selection, leading to a targeted expansion of specific bacterial clades.

#### 3.7.4. Functional Prediction of the Microbiome

To evaluate the potential metabolic and ecological functions, the Clusters of Orthologous Genes (COG) functional classification was performed (Figure 2D). Across all experimental groups, the predicted functional profiles were consistently enriched in basic metabolic and cellular processes. Excluding the uncharacterized “Others” category, the most abundant functional classifications were “Amino acid transport and metabolism” (10.0–11.3%), followed by “Energy production and conversion” (6.8–7.4%), “Translation, ribosomal structure and biogenesis” (6.6–7.2%), and “Inorganic ion transport and metabolism” (6.5–7.0%). Statistical analysis revealed no significant differences in the relative abundances of these COG functional categories among the five dietary treatments (*p* > 0.05). These results indicate that despite the structural and taxonomic shifts induced by CBD, the core functional homeostasis and overall metabolic stability of the intestinal microecosystem in *L. vannamei* were well preserved.

### 3.8. Median Lethal Dissolved Oxygen and Median Lethal Time (LT50) After Combined Stress

To evaluate the effect of dietary CBD on the environmental stress resistance of *L. vannamei*, the shrimp were subjected to a 4-day chronic stress of ammonia and nitrite (2 mg/L) and followed by an acute hypoxia challenge test. The median lethal dissolved oxygen (LDO_50_) and median lethal time (LT_50_) were determined under a combined stress challenge.

**Figure 3:**
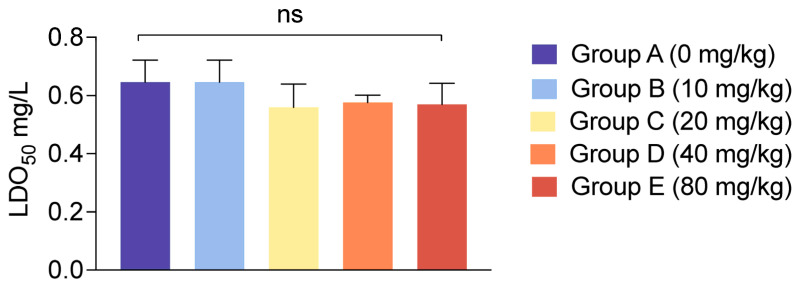
Effects of dietary CBD supplementation on the median lethal dissolved oxygen (LDO50) of *L. vannamei* under combined environmental stress. Shrimp were subjected to a 4-day chronic ammonia and nitrite stress followed by an acute hypoxia challenge. LDO50 values are presented as mean ± SD of 3 independent biological replicate buckets per treatment group. Groups A, B, C, D, and E correspond to CBD inclusion levels of 0, 10, 20, 40, and 80 mg/kg, respectively. ns, no statistically significant differences were observed among the dietary treatments determined by Duncan’s test (*p* > 0.05).

The results for LDO_50_ are detailed in Figure 3. There were no statistically significant differences in the LDO_50_ values among the five groups (*p* > 0.05). Across all treatments, mortality began to occur at DO levels between 0.51 and 0.72 mg/L, and the calculated LDO_50_ hovered consistently around 0.57 mg/L.

**Figure 4:**
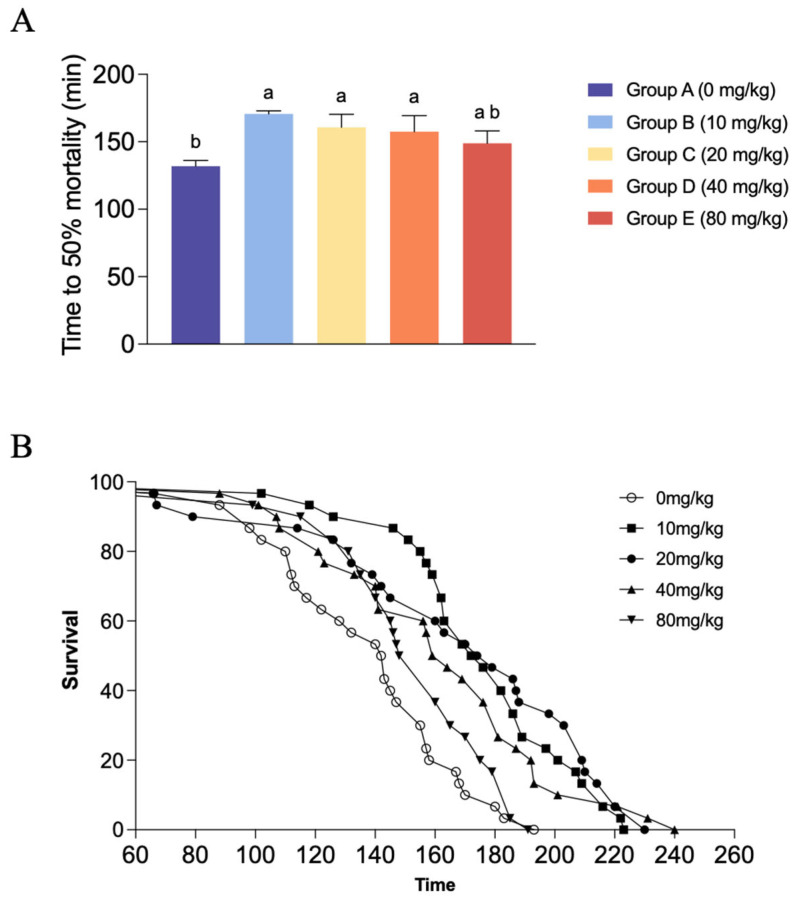
Effects of dietary CBD supplementation on the hypoxia tolerance of *L. vannamei* under combined environmental stress. (**A**) Median lethal time (LT50) across different dietary treatments during the acute hypoxia challenge. LT_50_ values are presented as mean ± SD of 3 independent biological replicate buckets per treatment group. Different lowercase letters indicate statistically significant differences among the groups (*p* < 0.05). Groups A, B, C, D, and E correspond to CBD inclusion levels of 0, 10, 20, 40, and 80 mg/kg, respectively. (**B**) Cumulative survival curves generated using the Kaplan–Meier method based on the death time of all 30 individual shrimp merged from 3 biological replicate buckets per treatment group, illustrating the rightward shift in survival trajectories for the CBD-supplemented groups.

In terms of LT_50_ (Figure 4A), the control group recorded the lowest average LT_50_ (131.94 ± 4.15 min). With the inclusion of dietary CBD at low to moderate levels, the LT_50_ values were significantly prolonged. Specifically, shrimp in Groups B, C, and D exhibited significantly higher LT_50_ values than those in the control group (*p* < 0.05), reaching a maximum of 170.63 ± 2.33 min in Group B. There were no significant differences in the LT_50_ values among these three moderate dosage groups (*p* > 0.05). However, as the CBD supplementation level further increased to 80 mg/kg (Group E), the LT_50_ value declined to 148.82 ± 9.21 min, which showed no significant statistical difference from either the control group or the other CBD-treated groups (*p* > 0.05). The cumulative survival curves further illustrate this trend, showing a clear rightward shift in the survival trajectories of the CBD groups (Figure 4B).

### 3.9. qPCR Analysis of Stress-Related Genes in the Hepatopancreas

**Figure 5:**
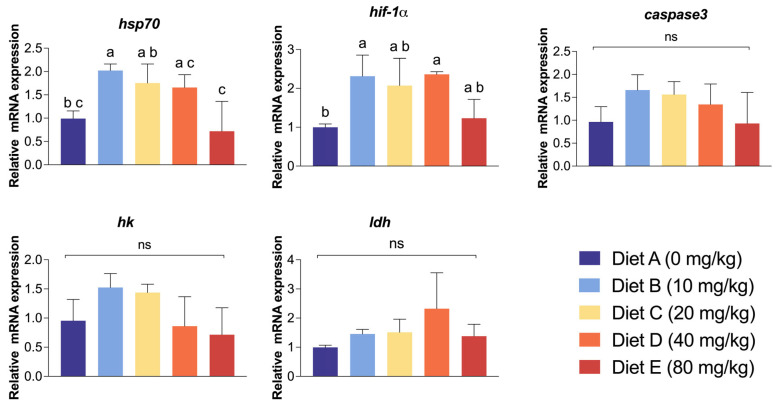
Relative mRNA expression levels of key stress, hypoxia, and metabolism-related genes in the hepatopancreas of *L. vannamei*. The quantified genes include heat shock protein 70 (*hsp70*), hypoxia-inducible factor 1-alpha (*hif-1α*), cysteine-aspartic acid protease 3 (*caspase3*), hexokinase (*hk*), and lactate dehydrogenase (*ldh*). Data are presented as mean ± SD (*n* = 3 per treatment). Different lowercase letters above the error bars denote statistically significant differences among the dietary treatments (*p* < 0.05), while “ns” indicates no significant difference. Groups A, B, C, D, and E correspond to CBD inclusion levels of 0, 10, 20, 40, and 80 mg/kg, respectively.

To evaluate the molecular response of the hepatopancreas to the combined stress challenge, the relative mRNA expression levels of key stress, hypoxia, and metabolism-related genes were quantified (Figure 5). The relative expression of *hsp70* exhibited a significant dose-dependent response. Its expression peaked in Group B (10 mg/kg), which was significantly higher than both the control group and the highest dosage group (*p* < 0.05). Conversely, Group E recorded the lowest *hsp70* expression level among all treatments. Similarly, the expression of *hif-1⍺* was significantly elevated. Groups B (10 mg/kg) and D (40 mg/kg) showed significantly higher *hif-1⍺* expression compared to the control (*p* < 0.05), while Groups C and E exhibited intermediate levels with no significant difference from the control. No significant differences in the expression levels of *ldh*, *caspase3*, and *hk* genes among any of the experimental groups (*p* > 0.05) were observed.

### 3.10. Correlation Analysis Among Growth, Physiology, and Stress Resistance

**Figure 6:**
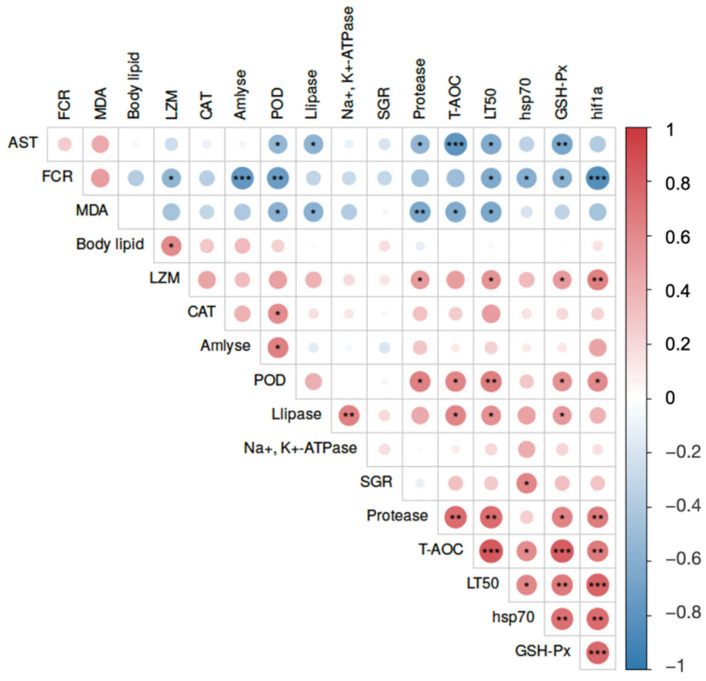
Pearson correlation matrix integrating macroscopic phenotypes and micro-physiological parameters in *L. vannamei*. The heatmap elucidates the potential internal relationships among core parameters of feed utilization (FCR), digestive capacity (protease, amylase), antioxidant status (T-AOC, GSH-Px, POD, MDA), hepatopancreatic health (AST), stress-related gene expression (*hif-1α*, *hsp70*), and ultimate environmental stress endurance (LT50). Color gradients represent the correlation coefficient (*r*), with positive correlations and negative correlations distinctly color-coded. Statistical significance for the correlation is defined as *, *p* < 0.05; **, *p* < 0.01; and ***, *p* < 0.001, respectively.

To elucidate the potential internal relationships between macroscopic phenotypes and physiological shifts, a Pearson correlation analysis was performed (Figure 6). The correlation matrix revealed robust, statistically significant interactions integrating feed utilization, antioxidant capacity, and ultimate stress endurance. Notably, the LT_50_ under combined stress exhibited strong positive correlations with the T-AOC (*r* = 0.842), *hif-1a* expression (*r* = 0.786), and protease activity (*r* = 0.748, *p* < 0.05). Conversely, LT_50_ was significantly and negatively correlated with the FCR, the oxidative damage marker MDA, and the hepatopancreatic injury indicator AST (*p* < 0.05). Furthermore, FCR was profoundly and negatively correlated with amylase activity (*r* = −0.764) and protective molecules like POD and *hsp70*, while positively correlating with MDA (*p* < 0.05). Within the physiological parameters, T-AOC showed highly synergistic positive correlations with GSH-Px and *hif-1a*, and strong negative correlations with AST and MDA (*p* < 0.05).

## 4. Discussion

Given the physiological challenges imposed by intensive farming practices, the exploration of bioactive phytochemicals to enhance shrimp health and productivity has become increasingly relevant. In this context, the present study evaluated the effects of CBD on *L. vannamei*, revealing dose-dependent responses in growth, immunity, and intestinal microecology.

### 4.1. Moderate CBD Improves Feed Efficiency and Muscle Fatty Acid Composition

Our findings demonstrate that moderate CBD supplementation effectively promotes somatic growth and minimizes the FCR in *L. vannamei*. These growth-promoting effects align closely with recent findings in various teleost models. For instance, dietary supplementation with cannabis extracts significantly improved the feed conversion and growth performance of Nile tilapia (*O. niloticus*) [22], while CBD administration enhanced appetite and somatic growth in juvenile large yellow croaker (*L. crocea*) fed high-lipid diets [27]. Rather than merely stimulating feed intake, our correlation matrix (Figure 6) highlights that the enhanced feed efficiency is fundamentally driven by the robust secretagogue effect of CBD on the hepatopancreas. By positively modulating the digestive enzymatic cascade—particularly amylase and protease activities—CBD facilitates the breakdown and apparent digestibility of dietary macromolecules. This is further corroborated by recent mammalian evidence showing that CBD profoundly regulates intestinal lipid absorption and metabolic transport [45].

Interestingly, CBD also exhibited a highly specific, bidirectional modulation of lipid metabolism. While the best somatic growth was observed at 40 mg/kg within the concentration range tested in this study, the lowest inclusion level (10 mg/kg) preferentially directed lipid fluxes toward the biosynthesis of HUFAs, maximizing the deposition of EPA and DHA in the muscle. This phenomenon is powerfully supported by recent biomedical research. For instance, Bielawiec et al. (2023) demonstrated that CBD administration significantly ameliorates muscular lipid profiles by inhibiting de novo lipogenesis and specifically altering the desaturation and elongation ratios of fatty acids in skeletal muscle [46]. Furthermore, CBD is well-documented to reduce intracellular lipid accumulation and prevent hepatosteatosis [47]. Taken together, these mechanistic insights suggest that low-dose CBD may act as a potent elicitor for endogenous lipid-modifying enzymes (i.e., elongases or desaturases) in crustaceans, diverting energy from simple fat storage toward the synthesis of essential structural lipids. This metabolic shift not only improves the physiological quality of the shrimp but also significantly elevates its nutritional value for human consumption.

### 4.2. CBD Enhances Environmental Stress Tolerance via Antioxidant and Hif-1α Pathways

Intensive shrimp farming is frequently compromised by cyclic hypoxia and ammonia accumulation, which provoke severe oxidative stress and tissue damage. The present study reveals that CBD significantly fortifies the environmental resilience of shrimp, effectively extending their survival window (LT_50_) under combined lethal stressors. Mechanistically, this survival advantage is deeply rooted in CBD’s intrinsic biochemical properties. The rich hydroxyl groups within the polyphenol-like structure of CBD confer exceptional free-radical scavenging capabilities [29,30]. Consequently, dietary CBD effectively neutralized ROS overproduction during hypoxia, preventing lipid peroxidation, as evidenced by suppressed MDA levels, and sparing endogenous antioxidant enzymes, such as GSH-Px and CAT, from rapid depletion. This robust systemic stress mitigation mirrors observations in teleosts, where CBD administration significantly attenuated cortisol levels and mitigated both acute and chronic stress responses in Nile tilapia [25,26].

Furthermore, our correlation network delineates a critical molecular pathway mediating this enhanced hypoxia tolerance: the up-regulation of the *hif-1α* signaling cascade. Strikingly, this phenotypic observation is strongly corroborated by a recent mammalian model study, which demonstrated that CBD administration effectively prevented antioxidant depletion, reduced lipid peroxidation, and notably stabilized HIF-1α protein levels during global hypoxia [48]. By sensitizing the *hif-1α* pathway prior to the acute hypoxic event, CBD enables the hepatopancreas of the shrimp to proactively assemble a robust antioxidant shield and heat shock protein chaperones [49]. This proactive defense mechanism minimizes structural damage to vital organs and sustains cellular energy production under extreme environmental duress.

### 4.3. CBD Modulates Intestinal Microbiota and Suppresses Opportunistic Pathogens

The gastrointestinal tract is not only the primary site for nutrient assimilation but also the foremost immunological barrier in crustaceans [50,51,52]. Our 16S rRNA sequencing revealed that moderate CBD supplementation exerted a targeted ecological selection pressure on the intestinal microbiota, positively restructuring the core community. Notably, the relative abundances of *Aeromonas* and *Pseudomonas*—classic opportunistic pathogens in aquaculture—were substantially suppressed in the low- to moderate-dose CBD groups [53]. This antimicrobial potential of CBD is highly consistent with recent in silico assessments, which identified that CBD exhibits potent inhibitory binding affinities against crucial structural proteins of severe aquatic pathogens [28].

Beyond direct molecular interactions, emerging biomedical research highlights CBD’s profound capacity to indirectly eliminate pathogens and protect the gut barrier via the host-microbiome axis. For instance, cannabinoid interventions have been shown to remodel the gut microecosystem by diminishing inflammation-associated opportunistic taxa (e.g., *Desulfovibrionaceae*) and promoting the intestinal secretion of antimicrobial peptides [34]. Furthermore, even when exposed to high pathogenic loads or bacterial endotoxins like lipopolysaccharides, CBD effectively antagonizes LPS-induced reactive enteric gliosis, intense intestinal inflammation, and epithelial apoptosis, thereby preserving the structural integrity of the mucosal barrier [34].

Concurrently, CBD-enriched core beneficial taxa, such as *Rhodobacteraceae*, are well documented for their roles in aquatic biogeochemical cycling and competitive pathogen exclusion [54,55]. This positive structural modulation of the microbiome likely alleviates the local inflammatory burden on the intestinal epithelium. Consequently, the host can reallocate energy resources to bolster systemic non-specific immunity, completely elucidating the significant elevation in serum lysozyme and phosphatase activities observed in our study.

Interestingly, the enhancement of baseline LZM activity by moderate CBD supplementation highlights its role as a proactive immune elicitor. In our study, CBD pre-conditioned the non-specific immune system of healthy shrimp prior to environmental challenges. This immunomodulatory behavior of CBD appears to be highly context-dependent. For instance, in a recent teleost model subjected to severe chronic stress, CBD exerted a stress-mitigating effect by reducing the abnormally spiked LZM levels back to physiological baselines [56]. Together with our findings, this dynamic contrast suggests that CBD functions not merely as a unidirectional immune stimulant, but rather as a profound homeostatic regulator, which bolsters immune readiness under normal conditions while preventing immune overexhaustion during severe stress.

### 4.4. Excessive CBD Supplementation Induces Metabolic Burden and Hepatopancreatic Toxicity

A critical objective of pharmacological and nutritional studies is identifying the safety threshold of novel additives. Our study revealed a classic biphasic dose–response effect of CBD in *L. vannamei*. While doses of 10–40 mg/kg conferred comprehensive physiological benefits within the range tested in this study, the highest dosage (80 mg/kg) aggressively compromised the host’s performance, leading to a deterioration in FCR and a diminished LT_50_ advantage.

This macroscopic decline was strictly mirrored by microscopic toxicological indicators, illuminating a severe metabolic burden. At 80 mg/kg, massive energy resources were seemingly diverted from somatic growth to the detoxification of excessive xenobiotics, drastically depleting whole-body lipid stores. More alarmingly, the serum ALT activity in the high-dose group surged to more than double that of the control, providing definitive evidence of increased hepatopancreatic membrane permeability and substantial tissue damage. Crucially, this finding aligns with recent toxicological warnings in mammalian models and clinical safety updates, where the administration of high-dose CBD has been explicitly linked to hepatotoxicity and drastic elevations in liver transaminases [57,58]. Furthermore, heavy or overdose applications of phytocannabinoids are increasingly recognized to provoke adverse systemic effects, overwhelming the host’s detoxification capacity and inducing severe metabolic exhaustion [59]. While low doses of CBD (10 and 20 mg/kg) efficiently enhance animal welfare and physiological homeostasis in Nile tilapia [25], the exhaustion of protective mechanisms at 80 mg/kg was evident in our study, characterized by the severe down-regulation of *hsp70* transcription and rebounding MDA levels. Collectively, these findings caution that while CBD is a highly promising functional additive, its dietary inclusion must be strictly regulated (recommended below 40 mg/kg based on the results of the present study) to prevent metabolic exhaustion and hepatopancreatic toxicity in intensive shrimp culture.

Despite the comprehensive physiological benefits observed, several limitations of the present study should be acknowledged. First, the 8-week feeding trial primarily focused on the juvenile stage of *L. vannamei*. Long-term studies encompassing the entire grow-out phase are required to fully evaluate the lifelong impacts and potential bioaccumulation of CBD. Second, the laboratory-controlled acute stress models may not completely replicate the complex, multifactorial stressors encountered in actual open-pond aquaculture systems, suggesting that large-scale field trials are necessary before commercial translation.

## 5. Conclusions

The present study establishes CBD as a highly promising functional feed additive for *L. vannamei*, characterized by a distinct biphasic dose–response. Moderate dietary CBD supplementation (10–40 mg/kg) significantly improves feed efficiency, enriches EPA and DHA in the muscle, and actively restructures the intestinal microecosystem by suppressing opportunistic pathogens within the concentration range tested in this study. Crucially, CBD supplementation at 10–40 mg/kg up-regulates *hif-1α* and *hsp70* pathways and amplifies systemic antioxidant networks, thereby dramatically extending survival under severe ammonia and hypoxia stress. However, excessive CBD inclusion (80 mg/kg) overwhelms the host’s detoxification capacity, resulting in lipid depletion, severe hepatopancreatic tissue damage, and compromised environmental resilience. Therefore, careful dosage regulation is imperative. Dietary CBD at the effective levels identified in this study (up to 40 mg/kg) represents a safe, sustainable, and powerful nutritional strategy to enhance growth, immune homeostasis, and stress endurance in intensive crustacean aquaculture.

## Figures and Tables

**Table 1:** Formulation and proximate analysis of the experimental diets (per 100g dry diet).

Ingredients (%)	Diet No. (CBD Level mg/kg)				
	Diet A (0 mg/kg)	Diet B (10 mg/kg)	Diet C (20 mg/kg)	Diet D (40 mg/kg)	Diet E (80 mg/kg)
Protein mixture ^a^	68	68	68	68	68
Wheat meal	13.8	13.8	13.8	13.8	13.8
α-starch	3	3	3	3	3
Cellulose	2.60	2.58	2.56	2.52	2.44
Fish oil	3	3	3	3	3
Soybean oil	1.5	1.5	1.5	1.5	1.5
Soy lecithin meal	1	1	1	1	1
Mineral premix ^b^	1.5	1.5	1.5	1.5	1.5
Vitamin premix ^c^	1	1	1	1	1
Ca(H_2_PO_4_)_2_·H_2_O	2	2	2	2	2
Choline chloride	0.3	0.3	0.3	0.3	0.3
Squid paste	2	2	2	2	2
Methionine	0.2	0.2	0.2	0.2	0.2
Y_2_O_3_	0.1	0.1	0.1	0.1	0.1
Hemp leaf powder (5% CBD)	0	0.02	0.04	0.08	0.16
Analyzed Composition (%)					
Crude protein	42.94	42.9	42.91	42.81	42.89
Crude lipid	6.46	6.49	6.48	6.48	6.47
Crude ash	9.31	9.37	9.34	9.35	9.38

^a^ The protein mixture consists of soybean meal (47.06%), fish meal (14.71%), peanut meal (11.76%), corn protein powder (11.76%), meat meal (5.88%), blood meal (4.41%), and brewer’s yeast (4.41%). ^b^ Contained the following per kilogram of mineral premix: Ca 10.5 g, Fe 1.0 g, Co 0.8 g, Se 0.02 g, Mg 12 g, Mn 3.8 g, K 90 g, Cu 3.0 g, Zn 10 g. ^c^ Contained the following per kilogram of vitamin premix: VA8000000 IU, VD3 2000000 IU, VE 50 g, VK 10 g, Thiamine hydrochloride 5 g; riboflavin 15 g, nicotinamide 40 g, calcium D-pantothenate 25 g, pyridoxine 8 g, cyanocobalamin 0.02 g, folic acid 2 g, biotin 0.08 g, inositol 100 g.

**Table 2:** Primers for qPCR analysis.

Gene Name		Nucleotide Sequence (5′-3′)	Sources
*hsp70*	Forward	AGGAGACCGCTGAGGCTTAC	DQ813343.1
	Reverse	AGCACATTCAGACCCGAGAT	
*caspase 3*	Forward	AGTTAGTACAAACAGATTGGAGCG	KC660103.1
	Reverse	TTGTGGACAGACAGTATGAGGC	
*hif-1α*	Forward	GGAGTCTTTGAGAGAGAG	FJ807918 [43]
	Reverse	GCCTCCTTCCGTGATCTTC	
*hk*	Forward	AGTCGCAGCAACAGGAAGTT	EF102106.1 [44]
	Reverse	CGCTCTTCTGGCACATGATA	
*ldh*	Forward	TTGTTACTGCTGGTGCTCGT	XM_070135849.1
	Reverse	AGCGACACTCAGTTTCTGGG	
*β-actin*	Forward	CTTGTGTGCGACAATGGCTC	XM_027364954.1
	Reverse	TCGATGGGGTACTTGAGGGT	

**Table 3:** Effects of dietary CBD levels on growth performance and feed utilization of *L. vannamei*.

Parameters	Diet A (0 mg/kg)	Diet B (10 mg/kg)	Diet C (20 mg/kg)	Diet D (40 mg/kg)	Diet E (80 mg/kg)
IBW (g)	0.52 ± 0.04	0.52 ± 0.04	0.52 ± 0.04	0.52 ± 0.04	0.52 ± 0.04
FBW (g)	15.51 ± 0.53 ^b^	17.09 ± 0.71 ^a^	16.9 ± 0.63 ^a^	16.71 ± 0.2 ^a^	16.78 ± 0.29 ^a^
WGR (%)	2883.24 ± 101.19 ^b^	3185.76 ± 136.16 ^a^	3150.31 ± 120.4 ^a^	3113 ± 37.66 ^a^	3127.37 ± 56.22 ^a^
FI (%/day)	4.18 ± 0.05 ^b^	4.28 ± 0.1 ^b^	4.46 ± 0.2 ^ab^	4.49 ± 0.18 ^ab^	4.62 ± 0.2 ^a^
SGR (%/day)	2.46 ± 0.02 ^b^	2.53 ± 0.03 ^a^	2.52 ± 0.03 ^a^	2.51 ± 0.01 ^a^	2.51 ± 0.01 ^a^
FCR	1.9 ± 0.03 ^a^	1.79 ± 0.02 ^b^	1.76 ± 0.04 ^b^	1.68 ± 0.01 ^c^	1.86 ± 0.04 ^a^
SV (%)	65.19 ± 3.39 ^c^	70.37 ± 1.28 ^bc^	74.81 ± 2.57 ^ab^	80.74 ± 5.13 ^a^	74.81 ± 4.63 ^c^

Note: Data were expressed as means ± S.D. (*n* = 3 replicate tanks per treatment). Data in the same row sharing different superscript letters denote significant differences determined by Duncan’s test (*p* < 0.05). Rows without superscript letters indicate no significant differences among treatments (*p* > 0.05). FBW: final body weight; FCR: feed conversion rate; FI: feed intake; IBW: initial body weight; SGR: specific growth rate; SV: survivability; WGR: weight gain rate.

**Table 4:** Proximate composition (% wet weight basis) of *L. vannamei* whole shrimp fed with experimental diets.

Parameters	Diet A (0 mg/kg)	Diet B (10 mg/kg)	Diet C (20 mg/kg)	Diet D (40 mg/kg)	Diet E (80 mg/kg)
Moisture	75.62 ± 0.37	75.71 ± 0.58	76.05 ± 0.61	76 ± 0.38	76.07 ± 0.48
Crude protein	18.88 ± 0.24	18.95 ± 0.15	18.67 ± 0.11	18.78 ± 0.09	18.78 ± 0.1
Total lipid	1.52 ± 0.03 ^b^	1.53 ± 0.01 ^b^	1.54 ± 0.05 ^b^	1.61 ± 0.09 ^a^	1.48 ± 0.1 ^c^
Ash	2.65 ± 0.1	2.55 ± 0.08	2.64 ± 0.11	2.47 ± 0.09	2.59 ± 0.25

Note: Diets were supplemented with increasing levels of CBD (0, 10, 20, 40, and 80 mg/kg diet). Data were expressed as means ± S.D. (*n* = 3 replicate tanks per treatment). Data in the same row sharing different superscript letters denote significant differences determined by Duncan’s test (*p* < 0.05). Rows without superscript letters indicate no significant differences among treatments (*p* > 0.05).

**Table 5:** Effects of dietary CBD levels on the muscle fatty acid composition of *L. vannamei*.

Fatty Acids	Diet A (0 mg/kg)	Diet B (10 mg/kg)	Diet C (20 mg/kg)	Diet D (40 mg/kg)	Diet E (80 mg/kg)
C14:0	0.22 ± 0.03	0.21 ± 0.01	0.22 ± 0.02	0.23 ± 0	0.22 ± 0.02
C15:0	0.13 ± 0.04 ^b^	0.14 ± 0.03 ^ab^	0.15 ± 0.02 ^ab^	0.17 ± 0.01 ^a^	0.14 ± 0.02 ^ab^
C16:0	15.2 ± 0.29	15.05 ± 0.22	15.1 ± 0.26	15.01 ± 0.2	15.21 ± 0.13
C17:0	0.82 ± 0.01 ^b^	0.86 ± 0.02 ^a^	0.81 ± 0.02 ^a^	0.8 ± 0.03 ^b^	0.64 ± 0.01 ^c^
C18:0	9.54 ± 0.18	9.63 ± 0.15	9.59 ± 0.18	9.44 ± 0.27	9.59 ± 0.16
C19:0	1.34 ± 0.09 ^b^	1.48 ± 0.1 ^ab^	1.42 ± 0.1 ^ab^	1.47 ± 0.11 ^ab^	1.55 ± 0.06 ^a^
C20:0	0.35 ± 0.02	0.32 ± 0.1	0.36 ± 0.03	0.37 ± 0.01	0.38 ± 0.01
∑SFA	27.6 ± 0.25	27.69 ± 0.51	27.65 ± 0.2	27.48 ± 0.59	27.73 ± 0.28
C16:1n-7	1.13 ± 0.09 ^a^	1.12 ± 0.03 ^a^	1.12 ± 0.12 ^a^	1.17 ± 0.06 ^a^	0.96 ± 0.09 ^b^
C17:1n-7	0.15 ± 0.05	0.17 ± 0.02	0.17 ± 0.03	0.18 ± 0.02	0.15 ± 0.07
C18:1n-9	20.13 ± 0.16 ^a^	18.69 ± 0.35 ^d^	19.18 ± 0.19 ^c^	19.51 ± 0.05 ^b^	19.56 ± 0.12 ^b^
C20:1n-9	1.4 ± 0.01 ^a^	1.3 ± 0.06 ^b^	1.41 ± 0.02 ^a^	1.41 ± 0.03 ^a^	1.4 ± 0.03 ^a^
C22:1n-9	0.38 ± 0.16 ^ab^	0.33 ± 0.09 ^b^	0.53 ± 0.13 ^a^	0.41 ± 0.08 ^ab^	0.42 ± 0.08 ^ab^
∑MUFA	23.2 ± 0.21 ^a^	21.61 ± 0.27 ^c^	22.42 ± 0.19 ^b^	22.68 ± 0.06 ^b^	22.48 ± 0.21 ^b^
∑n-7MUFA	1.28 ± 0.13 ^ab^	1.29 ± 0.04 ^ab^	1.29 ± 0.10 ^ab^	1.35 ± 0.08 ^a^	1.11 ± 0.06 ^b^
∑n-9MUFA	21.91 ± 0.15 ^a^	20.32 ± 0.31 ^c^	21.12 ± 0.13 ^b^	21.33 ± 0.02 ^b^	21.37 ± 0.20 ^b^
C18:2n-6 (LA)	18.92 ± 0.18 ^a^	16.78 ± 0.23 ^c^	18.01 ± 0.24 ^b^	19.02 ± 0.25 ^a^	18.26 ± 0.15 ^b^
C18:3n-3 (LNA)	1.36 ± 0.03 ^ab^	1.21 ± 0.11 ^c^	1.31 ± 0.03 ^ab^	1.38 ± 0.05 ^a^	1.28 ± 0.03 ^bc^
C20:2n-6	2.08 ± 0.07 ^a^	1.95 ± 0.03 ^b^	2.1 ± 0.05 ^a^	2.03 ± 0.01 ^ab^	2.09 ± 0.08 ^a^
C20:4n-6 (ARA)	1.96 ± 0.03 ^a^	2.22 ± 0.06 ^b^	1.94 ± 0.12 ^a^	2.01 ± 0.08 ^a^	2.02 ± 0.03 ^a^
C20:5n-3 (EPA)	13.47 ± 0.29 ^cd^	15.22 ± 0.16 ^a^	13.77 ± 0.05 ^b^	13.21 ± 0.18 ^d^	13.72 ± 0.07 ^bc^
C22:5n-3	0.67 ± 0.02 ^b^	0.7 ± 0.03 ^ab^	0.72 ± 0.03 ^a^	0.71 ± 0.02 ^ab^	0.69 ± 0.02 ^ab^
C22:6n-3 (DHA)	10.74 ± 0.21 ^d^	12.62 ± 0.16 ^a^	12.08 ± 0.35 ^b^	11.49 ± 0.21 ^c^	11.73 ± 0.17 ^c^
∑PUFA	49.2 ± 0.32 ^c^	50.7 ± 0.39 ^a^	49.93 ± 0.33 ^b^	49.84 ± 0.64 ^b^	49.79 ± 0.2 ^ab^
∑n-6PUFA	22.96 ± 0.2 ^b^	20.95 ± 0.28 ^c^	22.05 ± 0.21 ^b^	23.06 ± 0.25 ^a^	22.38 ± 0.22 ^b^
∑n-3PUFA	26.24 ± 0.44 ^d^	29.75 ± 0.26 ^a^	27.88 ± 0.43 ^b^	26.78 ± 0.39 ^c^	27.41 ± 0.17 ^b^
n-3/n-6PUFA	1.14 ± 0.03 ^d^	1.42 ± 0.02 ^a^	1.26 ± 0.03 ^b^	1.16 ± 0.01 ^c^	1.23 ± 0.02 ^b^

Note: Data are expressed as mean ± SD (*n* = 4). Data in the same row sharing different superscript letters denote significant differences determined by Duncan’s test (*p* < 0.05). Rows without superscript letters indicate no significant differences among treatments (*p* > 0.05). Only the main fatty acids are listed in the table, including: C14:0, C15:0, C16:0, C18:0, C19:0, C20:0, C16:1n-7, C17:1n-7, C18:1n-9, C20:1n-9, C22:1n-9, C18:2n-6 (LA), C18:3n-3 (LNA), C20:2n-6, C20:4n-6 (ARA), C20:5n-3 (EPA), C22:5n-3, and C22:6n-3 (DHA). ΣSFA: saturated fatty acids; ΣMUFA: monounsaturated fatty acids; ΣPUFA: polyunsaturated fatty acids.

**Table 6:** Effects of dietary CBD levels on hepatopancreatic digestive enzyme activities and apparent digestibility of nutrients in *L. vannamei*.

Parameters	Diet A	Diet B	Diet C	Diet D	Diet E
	(0 mg/kg)	(10 mg/kg)	(20 mg/kg)	(40 mg/kg)	(80 mg/kg)
Digestive Enzymatic Activity					
Amylase/(U/gprot)	0.98 ± 0.04 ^d^	1.01 ± 0.02 ^cd^	1.03 ± 0.01 ^bc^	1.16 ± 0.03 ^a^	1.06 ± 0.02 ^b^
Protease/(U/gprot)	2841.73 ± 34.97 ^b^	2965.67 ± 30.64 ^a^	2931.04 ± 37.35 ^a^	2930.44 ± 44.15 ^a^	2924.97 ± 24.09 ^a^
Lipase/(U/gprot)	2.19 ± 0.49 ^c^	4.22 ± 1.29 ^ab^	5.03 ± 1.04 ^a^	3.22 ± 0.65 ^bc^	2.85 ± 0.57 ^c^
Na^+^, K^+^-ATPase/(U/mgprot)	23.57 ± 0.49 ^b^	24.27 ± 0.74 ^b^	27.15 ± 1.53 ^a^	23.88 ± 0.65 ^b^	23.74 ± 1.01 ^b^
Apparent Digestibility					
Dry matter	81.53 ± 1.77 ^b^	81.71 ± 1.33 ^b^	84.83 ± 1.48 ^a^	82.5 ± 0.84 ^ab^	81.53 ± 2.53 ^b^
Crude protein	86.96 ± 1.25 ^b^	87.03 ± 0.94 ^b^	89.31 ± 1.04 ^a^	88.07 ± 0.57 ^ab^	87.14 ± 1.76 ^b^
Crude lipid	89.84 ± 0.98 ^b^	92.45 ± 0.55 ^a^	92.93 ± 0.69 ^a^	90.5 ± 0.45 ^b^	89.08 ± 1.5 ^b^

Note: Data are expressed as mean ± SD (*n* = 4). Data in the same row sharing different superscript letters denote significant differences determined by Duncan’s test (*p* < 0.05).

**Table 7:** Effects of dietary CBD levels on serum biochemical parameters of *L. vannamei*.

Parameters	Diet A	Diet B	Diet C	Diet D	Diet E
	(0 mg/kg)	(10 mg/kg)	(20 mg/kg)	(40 mg/kg)	(80 mg/kg)
ALT/(U/L)	1.69 ± 1.09 ^b^	2.09 ± 1.31 ^b^	1.88 ± 0.53 ^b^	1.61 ± 0.38 ^b^	3.72 ± 0.46 ^a^
AST/(U/L)	32.69 ± 2.55 ^a^	20.93 ± 2.46 ^b^	29.46 ± 1.18 ^a^	29.33 ± 1.86 ^a^	30.31 ± 2.02 ^a^
LZM/(U/mL)	164.06 ± 23.59 ^b^	220.31 ± 38.65 ^ab^	206.24 ± 15.31 ^ab^	224.99 ± 26.52 ^a^	178.12 ± 24.21 ^ab^
ALP/(U_KA/100 mL)	8.38 ± 0.35 ^c^	9.48 ± 0.51 ^b^	10.94 ± 0.94 ^a^	10.84 ± 0.49 ^a^	9.23 ± 0.91 ^bc^
ACP/(U_KA/100 mL)	13.49 ± 0.37 ^b^	12.93 ± 0.27 ^b^	14.85 ± 0.55 ^a^	14.94 ± 0.56 ^a^	13.32 ± 0.29 ^b^
T-AOC/(mM)	0.34 ± 0.02 ^c^	0.43 ± 0.01 ^b^	0.49 ± 0.01 ^a^	0.35 ± 0.01 ^c^	0.3 ± 0.01 ^d^
CAT/(U/mL)	1.71 ± 0.23 ^b^	1.87 ± 0.38 ^ab^	1.82 ± 0.11 ^b^	2.21 ± 0.19 ^a^	1.75 ± 0.21 ^b^
MDA/(mmol/L)	4.62 ± 0.57 ^a^	3.94 ± 0.25 ^b^	3.71 ± 0.62 ^b^	3.86 ± 0.38 ^b^	4.85 ± 0.25 ^a^

Note: Data are expressed as mean ± SD (*n* = 4). Data in the same row sharing different superscript letters denote significant differences determined by Duncan’s test (*p* < 0.05).

**Table 8:** Effects of dietary CBD levels on hepatopancreatic antioxidant-related parameters in *L. vannamei*.

Parameters	Diet A	Diet B	Diet C	Diet D	Diet E
	(0 mg/kg)	(10 mg/kg)	(20 mg/kg)	(40 mg/kg)	(80 mg/kg)
T-AOC/(mmol/gprot)	1.41 ± 0.06 ^c^	2.04 ± 0.14 ^a^	1.71 ± 0.09 ^b^	1.71 ± 0.08 ^b^	1.58 ± 0.1 ^b^
CAT/(U/mgprot)	3.5 ± 0.46 ^b^	4.37 ± 0.57 ^a^	4.34 ± 0.36 ^a^	4.59 ± 0.65 ^a^	4.26 ± 0.57 ^ab^
GSH-Px/(U/mgprot)	1637.75 ± 41.77 ^c^	1853.55 ± 78.09 ^a^	1731.24 ± 38.79 ^b^	1739.48 ± 43.71 ^b^	1674.25 ± 53.04 ^bc^
POD/(U/mgprot)	21.58 ± 3.17 ^b^	31.06 ± 4.37 ^a^	28.66 ± 3.36 ^a^	33.51 ± 3.54 ^a^	27.83 ± 2.99 ^ab^
MDA/(nmol/mgprot)	26.7 ± 0.73 ^a^	23.15 ± 0.62 ^b^	22.38 ± 0.86 ^b^	23.12 ± 1.04 ^b^	22.81 ± 0.23 ^b^

Note: Data are expressed as mean ± SD (*n* = 4). Data in the same row sharing different superscript letters denote significant differences determined by Duncan’s test (*p* < 0.05).

## Data Availability

The original contributions presented in this study are included in the article. Further inquiries can be directed to the corresponding authors.

## Abbreviations

The following abbreviations are used in this manuscript:

ACP	Acid phosphatase
ADDM	Apparent Dry Matter Digestibility
ALP	Alkaline phosphatase
ALT	Alanine aminotransferase
AND	Apparent Nutrient Digestibility
ANOVA	Analysis of variance
ARA	Arachidonic acid
AST	Aspartate aminotransferase
*caspase 3*	Cysteine-aspartic acid protease 3
CAT	Catalase
CBD	Cannabidiol
COG	Clusters of Orthologous Genes
DHA	Docosahexaenoic acid
DO	Dissolved oxygen
EPA	Eicosapentaenoic acid
FBW	Final body weight
FCR	Feed Conversion Ratio
FI	Feed intake
GLU	Glucose
GSH-Px	Glutathione peroxidase
*hif-1α*	Hypoxia-inducible factor 1-alpha
*hk*	Hexokinase
*hsp70*	Heat shock protein 70
HUFA	Highly unsaturated fatty acids
IBW	Initial body weight
LA	Linoleic acid
LDA	Linear discriminant analysis
LDO50	Median lethal dissolved oxygen
*ldh*	Lactate dehydrogenase
LEfSe	Linear Discriminant Analysis Effect Size
LNA	Linolenic acid
LPS	Lipopolysaccharides
LT50	Median lethal time
LZM	Lysozyme
MDA	Malondialdehyde
OTUs	Operational Taxonomic Units
PC1/PC2	Principal coordinates
PCoA	Principal Coordinates Analysis
POD	Peroxidase
ROS	Reactive oxygen species
SD	Standard deviation
SGR	Specific Growth Rate
SV	Survivability
T-AOC	Total antioxidant capacity
TG	Triglycerides
THC	Δ9-tetrahydrocannabinol
WGR	Weight Gain Rate
ΣMUFA	Monounsaturated fatty acids
ΣPUFA	Polyunsaturated fatty acids
ΣSFA	Saturated fatty acids
